# Seroprevalence and risk factors for bovine brucellosis in the Chittagong Metropolitan Area of Bangladesh

**DOI:** 10.1002/vms3.348

**Published:** 2020-09-19

**Authors:** Shariful Islam, Shama Ranjan Barua, Shahnaj Parvin Moni, Ariful Islam, A. K. M. Anisur Rahman, Sharmin Chowdhury

**Affiliations:** ^1^ Chittagong Veterinary and Animal Sciences University (CVASU) Chittagong Bangladesh; ^2^ Institute of Epidemiology Disease Control and Research (IEDCR) Dhaka Bangladesh; ^3^ Ecohealth Alliance New York NY USA; ^4^ Department of Livestock Services Farmgate Dhaka Bangladesh; ^5^ Centre for Integrative Ecology, School of Life and Environmental Science Deakin University Geelong VIC Australia; ^6^ Bangladesh Agricultural University Mymensingh Bangladesh

**Keywords:** brucellosis, cELISA, lactating cows, RBPT, seroprevalence

## Abstract

Brucellosis is a neglected endemic zoonosis in Bangladesh and has a significant impact on public health and animal welfare of dairy farming as well as dairy farm economics. A cross‐sectional study was conducted to evaluate the seroprevalence of and risk factors for brucellosis in dairy cattle in the Chittagong metropolitan area (CMA) of Chittagong, Bangladesh. We collected serum samples (*n* = 158) from six randomly selected dairy farms in the CMA between February and November, 2015. The Rose Bengal Plate Test (RBPT) and a competitive ELISA (cELISA) were used as the screening and confirmatory tests respectively. Farm level and animal level demographic and risk factor data were collected using a questionnaire. The risk factors were analysed using a multivariable logistic regression with random effects. The overall seroprevalences of antibodies against brucellosis in cattle were 21.5% (34/158) and 7.6% (12/158) based on parallel and serial interpretation of the two tests respectively. Our results revealed that 20.3% (32/158) samples were positive using the RBPT and 8.9% (14/158) were positive using the cELISA. The within‐herd seroprevalence ranged from 10% to 26.3% and 5 to 20.7% using the RBPT and cELISA tests respectively. The odds of seropositivity were significantly higher in lactating cows (OR: 2.59; 95% CI: 1.02–6.55), cows producing less than 2 litres of milk (OR: 29.6; 95% CI: 4.3–353.8), cow producing 2–12 litres of milk (OR: 4.8; 95% CI: 1.1–33.4) and cows with reproductive disorders (OR: 3.2; 95% CI: 1.2–10.1). About 7.6% (12/158) and 1.3% (2/158) of cattle were found to be infected with acute and chronic brucellosis respectively. Based on these results, we suggest that cows that have reproductive disorders and are producing little milk should be prioritized for brucellosis screening in CMA. The screening tests should be used to control brucellosis in cattle in order to protect animal welfare, human health and to minimize the economic losses.

## Graphical abstract

A cross‐sectional study was conducted to evaluate the seroprevalence of and risk factors for brucellosis in dairy cattle in the Chittagong metropolitan area (CMA) of Chittagong, Bangladesh. The overall seroprevalences of antibodies against brucellosis in cattle were 21.5% (34/158) and 7.6% (12/158) based on parallel and serial interpretation of the two tests, respectively. Based on these results, we suggest that cows that have reproductive disorders and are producing little milk should be prioritized for brucellosis screening in CMA.

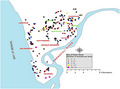

## 1 INTRODUCTION

Brucellosis is an important disease impacting veterinary and public health worldwide and caused by bacteria of the genus *Brucella* (Deka, Magnusson, Grace, & Lindahl, 2018). Globally, it is the second most frequently reported zoonotic disease to the World Organization for Animal Health (OIE) as it is regarded the most devastating trans‐boundary animal diseases, which cause significant trade obstructions (OIE, 2020; WHO, 2015). In domestic ruminants, brucellosis can cause reduced fertility, abortion, poor weight gain, lost draught power and a substantial decline in milk production (Franc, Krecek, Häsler, & Arenas‐Gamboa, 2018). In humans, it is considered to be an occupational disease among those that handle domestic ruminants affecting farmers, slaughter‐house workers, butchers, and veterinarians (Jimale, 2018; Kosgei, 2016; Mangalgi, Sajjan, Mohite, & Gajul, 2016; Mutua, 2017). The causative agent, *Brucella abortus*, can be transmitted to people through inhalation, contact with animal fluids, and consumption of unpasteurized dairy products and under‐cooked meat products (Jimale, 2018; Kosgei, 2016; Mutua, 2017; Olsen & Palmer, 2014).

Brucellosis is endemic in both humans and animals in Bangladesh (Rahman et al., 2017). Previous studies estimate the overall seroprevalence of brucellosis in cattle to be 2.4%–18.4%, while the herd‐level seroprevalence in cattle was estimated as 62.5% in Bangladesh (Ahasan, Rahman, & Song, 2010; Belal & Ansari, 2013; Nahar & Ahmed, 2009; Rahman et al., 2011; Rahman et al., 2019; Sikder et al., 2012). More precisely, seroprevalence was estimated to be 5% in Chittagong, Bangladesh (Sikder et al., 2012).

The diagnosis of brucellosis continues to be challenging in developing countries like Bangladesh. Rose Bengal Plate Test (RBPT) is the most commonly used conventional screening test for brucellosis in animals (Musallam, Abo‐Shehada, Omar, & Guitian, 2015; Rahman, 2015). RBPT relies on the unique antigenic properties of lipopolysaccharides (LPS) that are present within the cell membrane of *Brucella* spp.; however, the LPS antigen is also present in a number of other gram negative bacteria, including *Vibrio* and *Yersinia enterocolitica*, which may cross‐react on the brucellosis diagnostic assays (Munoz et al., 2005). The OIE recommends the use of Competitive Enzyme Linked Immunosorbent Assay (cELISA) for international cattle trade as this assay is reported to be more specific than RBPT (OIE, 2016; Rahman et al., 2019; Wang et al., 2015).

Chittagong District is one of the most important intensive dairy production regions in Bangladesh with 109–175 cattle/kmsq (Huque & Khan, 2017). However, only one previous study reported the seroprevalence and risk factors for brucellosis from Chittagong district (Sikder et al., 2012). Our objectives were to estimate the seroprevalence of brucellosis and to identify the risk factors for brucellosis in dairy cattle of Chittagong Metropolitan Area, Bangladesh.

## 2 MATERIALS AND METHODS

### 2.1 Ethics statement

Informed verbal consent was obtained from all animal owners for the collection of blood samples from their cattle (Appendix S1). This study was approved by the animal Ethics Committee of Chittagong Veterinary and Animal Sciences University, Bangladesh (Approval no: EC/2014/34‐7).

### 2.2 Study area, design and target population

**FIGURE 1:**
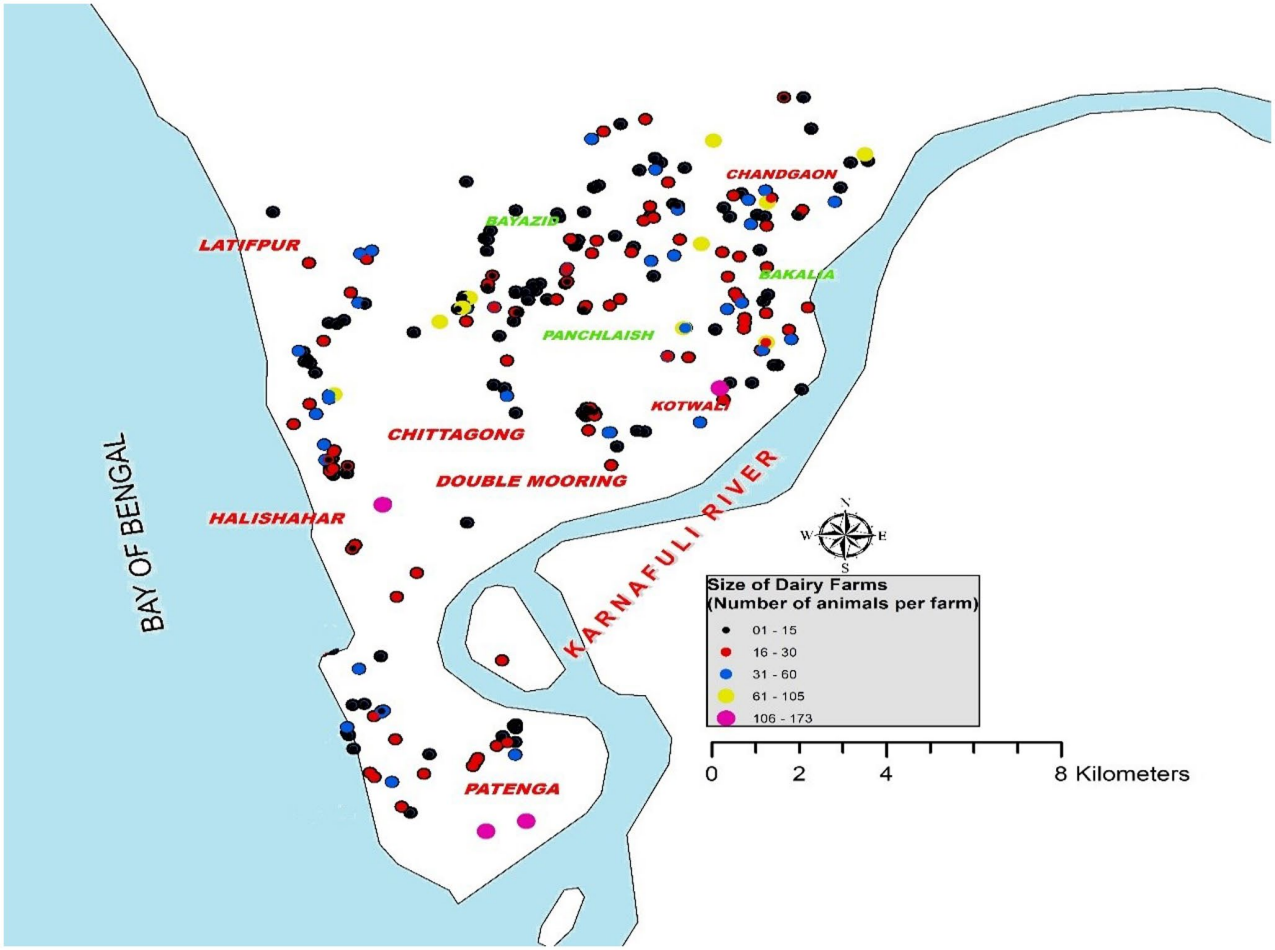
Distribution of cattle farms in Chittagong Metropolitan Area (*n* = 293)

This study was conducted in dairy cattle in the Chittagong metropolitan area (CMA), Bangladesh (22°22'N and 91°48'E) from February to November 2015. The study area is situated in the tropical zone and characterized by an annual average range temperature of 13–32°C, 5.6–727 mm of rainfall and 70%–85% humidity of (Anon, 2016). Chittagong metropolitan has 14 distinct areas in five sub‐regions like Chandgoan (North), Bayazid (West), Bakalia (East), Halishahar (South) and Panchlaish (Central) (Figure 1). We conducted a cross sectional study including all of the five sub‐regions. Moreover, we have identified the list of all of the available commercial cattle farms from each sub‐region. Six farms were randomly selected from the farm list where one farm from each sub‐region except Panchlaish where two farms were chosen. Within the study area in the CMA there were 293 cattle farms (Figure 1) including 57 commercial dairy farms that keep 3,084 cattle (Table 1). A farm was considered commercial if they had at least 25 cattle on the farm. The distribution of farms and cattle population by study sub‐sites is presented in Table 1.

**TABLE 1:** Distribution of farms and cattle population by study sub‐sites in Chittagong Metropolitan areas, Chittagong

Study sub‐sites	Number of farms in CMA	Total cattle population in CMA	Range of number of cattle per farm in CMA
Chandgaon (North)	21	748	3–93
Bayazid (West)	29	263	5–43
Bakalia (East)	40	683	11–135
Halishahar (South)	15	806	4–80
Panchlaish (Centre)	39	584	4–45

### 2.3 Sample size determination

The required sample size (n) to estimate seroprevalence with 95% confidence was calculated based on the equation 1 (Charani et al., 2010).

$$n=\frac{1.96^{2}\times P\times \left(1‐P\right)}{d^{2}}$$

where P is the expected seroprevalence (10%) and d is the desired precision (5%). We calculated a minimum required sample size of 138.

### 2.4 Sampling strategy and distribution of sampled farms and cattle population

Within the farm, we could not collect samples from every animal due to some problem with technical and proper restraining of animals. Moreover, for few animals, the owner did not allow to collect the samples, as they are in advance stage pregnancy. Thereafter, all eligible cattle (158) belonging to the selected farms were sampled and almost matched up with the estimated sample size (*n* = 158) for the study. The distribution of sampled farms and population are presented in Table 2.

**TABLE 2:** Distribution of sampled farms and eligible population (only mature dairy cattle) by study sub‐sites in Chittagong Metropolitan areas, Chittagong (*N* = 6 farms, *n* = 158 cattle)

Study sub‐sites	No. of selected farm	Total eligible population	Total no. of samples collected
Chandgoan (North)	1	37	29
Bayazid (West)	1	51	39
Bakalia (East)	1	33	29
Halishahar (South)	1	25	20
Panchlaish (Centre)	2	47	41
Total	6	193	158

### 2.5 Collection of cattle blood samples and recording epidemiological data

To collect the blood samples, the cattle were run through a chute and held with a head gate. We conducted the venipuncture using the jugular vein and collected 5–10 ml of blood using serum tubes. The blood sample was labelled using the tag number assigned to each individual animal. The tubes were held vertically at room temperature for 1 hr and were then refrigerated at 4°C overnight before spinning at 3,000 rpm (2,555 g) for 10 min. The separated sera samples were placed into sterile Eppendorf tubes and kept in a freezer (−20°C) until tested.

A standard questionnaire was administered orally to each farmer. This questionnaire examined animal‐ and farm‐level risk factors. The investigated animal‐level risk factors included age, parity, breed, lactation stage, body condition score (BCS), history of reproductive issues and milk yield. We also asked about the following general characteristics about the farm, the management system, recording system and biosecurity measures. The questionnaire data were obtained by visual examination of the farm, directly reviewing the records and by asking the farmer. We estimated the variables by physical examination of the animal (e.g. age of the animal, pregnancy period of the animal, etc.). To validate data regarding milk yield per animal, farms were visited during the time of milking. Body condition scores were assessed by using the following characteristics: the amount of muscling and fat deposition over and around the vertebrae in the loin region of the cows (Stádník & Atasever, 2015). The score was scaled from 1 to 5 (1 = emaciated; 2 = thin; 3 = average; 4 = fatty; and 5 = obese cows).

The questionnaire was administered to one member of the farm who was knowledgeable about the herd. The information collected included retrospective information and each interview took about 30–40 min. The geographic location of each farm was recorded using a hand‐held Garmin^®^ Global Positioning Systems (GPS) (Garmin, 1,200 East 151st Street Olathe, KS 66062‐3426 USA).

### 2.6 Laboratory evaluation

Two serological tests were used to evaluate serum samples for brucellosis in cattle, the RBPT (Rahman, 2015) and cELISA (Chikweto et al., 2013).

#### 2.6.1 Rose Bengal plate test

The RBPT was conducted using the Atlas *Brucella* test^®^ (Atlas Medical, Cambridge), which is a rapid agglutination test, and was conducted per the manufacturer's instructions. The sensitivity and specificity of RBPT for the diagnosis of bovine brucellosis in Bangladesh were reported to be 87.4% and 99.4% respectively (Rahman et al., 2019). Briefly, equal volume (30 µl) of RBPT reagent and serum were mixed and rotated on a glass slide for 1 min. The result was considered positive if visible agglutination was identified positive and negative control sera samples used during RBPT were collected from Department of Medicine laboratory, Bangladesh Agricultural University.

#### 2.6.2 Enzyme linked immunosorbent assay

The SVANOVIR *Brucella*‐Ab cELISA was used following manufacturer's instructions (SVANOVA^®^, article no: 104893, Boehringer Ingelheim Svanova Box 1545 SE‐751 45 Uppsala, Sweden) (Chikweto et al., 2013). The sensitivity and specificity of the C‐ELISA as reported by the manufacture are 98% and 99.7% respectively (SVNOVIR®Brucella C‐ELISA (Svanova, 2020). Briefly, within 15 min after addition of stop solution, the optical density (OD) was measured at 450 nm for each of the controls and serum samples using a microplate ELISA reader. The positive or negative cut‐off was calculated at 30% inhibition (PI). Any test sample giving PI equal to or above this value was regarded as positive. The kit is designed to detect antibodies to *B. abortus* and *B. melitensis* in serum. In cattle, the assay is capable of distinguishing between *Brucella* infected animals, *Brucella* strain 19 vaccinated animals and animals infected with cross‐reacting gram negative bacteria.

### 2.7 Data entry and statistical analysis

Field and laboratory data were cleaned and coded in MS Excel 2007 before exporting to STATA‐13 (*StataCrop*) for epidemiological analysis.

#### 2.7.1 Descriptive analysis

Categorical variables were summarized as frequency, percentages and 95% confidence intervals (95% CI); and continuous variables were summarized as mean ± standard deviation (*SD*). An animal was considered seropositive if it is tested positive to either RBPT or cELISA (parallel interpretation). A herd, defined as the total number of cattle belonging to the same household, was considered seropositive if it included at least one seropositive animal. Animal and herd apparent seroprevalences were calculated by dividing the number of positive test results by the total number of animals and herds sampled respectively. The within‐herd seroprevalence was calculated by dividing the number of seropositive animals in the herd by the total number of animals tested in that herd. We calculated 95% CIs for all seroprevalence estimates.

**FIGURE 2:**
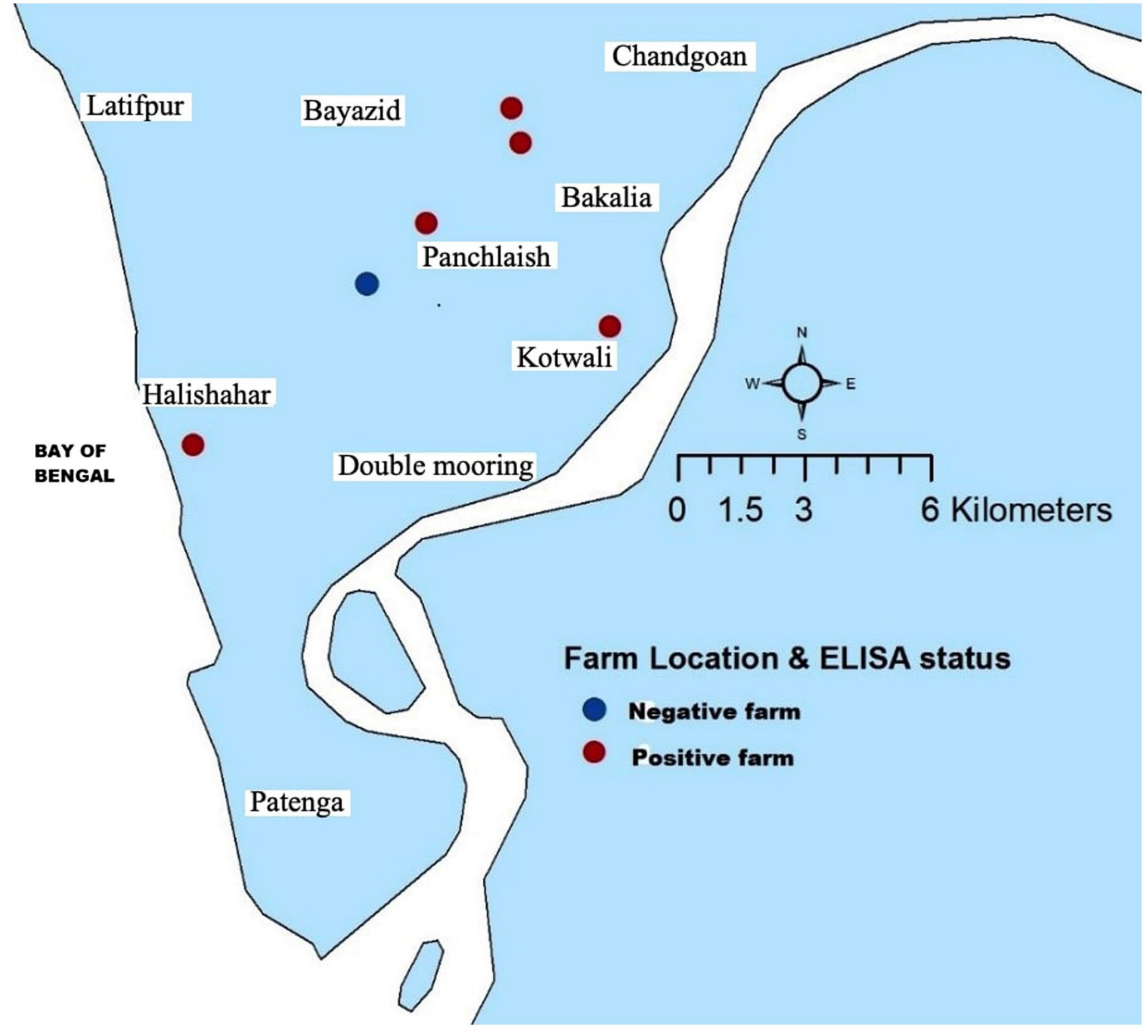
Geographical location and infection status of the farms by cELISA

Dot maps were created to show the location of the study population, the sampled farms and the herd sero‐status (positive and negative) based on the result of cELISA were shown in dot maps. The dot maps were created by ArcGIS version 10.2.1 (ESRI) was used (Figures 1 and 2).

#### 2.7.2 Risk factor analysis

##### Univariable analysis

The associations between seropositivity and categorical risk factors were tested using univariable random effects (RE) logistic regression analysis considering farm as random effect.

##### Multivariable analysis

Independent variables that were significantly associated with seropositivity in the univariable analysis (*p* < .20) were included in to the multivariable RE logistic regression model. In the multivariable analysis, a backward elimination procedure was used applying the maximum likelihood estimation procedure and statistical significance of contribution of individual predictors (or group of predictors) to the models tested using the Wald's test and likelihood ratio test (Dohoo, Martin, & Stryhn, 2003). Regression coefficients were converted into odds ratios (ORs) and their 95% confidence intervals (CIs). The interactions between variables were assessed by constructing two‐way interaction product terms for the significant main effect variables in the model, forcing them into the model and examining changes in the odds ratio (OR) and p values of the main effects. Confounding effect of the explanatory variables was evaluated by observing the change of parameter estimates before and after removal of a variable from the model. If the parameter estimate of a variable increased or decreased ≥ 15% after removing a variable from the model, then that explanatory variables was considered to have confounding effect on the outcome variable. All biologically meaningful interactions were also assessed.

### 2.8 Comparison of serological tests

The seroprevalence of bovine brucellosis was compared between RBPT and cELISA using Kappa statistics to identify the test agreement and the test characteristics of RBPT were calculated considering cELISA as gold standard. The kappa (κ) value was interpreted as one of the following: poor (κ = 0), slight (0.01 < κ < 0.20), fair (0.21 < κ < 0.40), moderate (0.41 < κ < 0.60), almost perfect (0.61 < κ < 0.80) and excellent (0.81 < κ < 1.00) (Carrouel et al., 2016).

## 3 RESULTS

### 3.1 Overall seroprevalence of brucellosis in cattle

The overall seroprevalences of bovine brucellosis in cattle were 21.5% (34/158) and 7.6% (12/158) based on parallel and serial interpretation of the two tests respectively. The seroprevalences using the RBPT and cELISA assays were 20.3% (95% CI: 14–27; *N* = 158) and 8.9% (95% CI: 5–14; *N* = 158) respectively. The difference in the results between the two diagnostic assays varied significantly (*χ* ^2^ 40.75; *p* = .000). All farms (*N* = 6) were seropositive by RBPT whereas five farms were seropositive by cELISA (Figure 2). The within‐herd seroprevalence of brucellosis ranged from 10% to 26.3% and 5 to 20.7% by RBPT and cELISA respectively (Table 3).

**TABLE 3:** The within‐herd seroprevalence of brucellosis in dairy cattle farms in the Chittagong metropolitan area

Study sub‐sites		RBPT Prevalence (95% CI)	cELISA % positive (95% CI)
Chandgaon (North)	29	24.1 (10–43)	20.7 (8–40)
Bayazid (West)	39	20.5 (9–36)	5.1 (6–17)
Bakalia (East)	29	20.7 (8–40)	6.9 (1–22)
Halishahar (South)	20	10.0 (1–32)	5.0 (0.1–25)
Panchlaish (Centre)	19	26.3 (9–51	0 (0, 18)^a^
Panchlaish (Centre)	22	18.2 (5–40)	13.6 (3–35)

^a^ 97.5% CI.

The herd sero‐status based on cELISA result is presented in Figure 2. One farm had no cattle that tested seropositive on the cELISA (Panchlaish).

### 3.2 Risk factors

#### 3.2.1 Univariable analysis

In Fisher's exact test and univariable logistic regression analyses, lactation, anestrous, a history of reproductive disorders, milk yield, lactation number, trimester and abortion were significantly (*p* ≤ .2) associated with brucellosis seropositivity (Table 4).

**TABLE 4:** Univariate associations between potential risk factors and brucellois in dairy cattle of CMA, Bangladesh

Variables	Category	Tested		Univariable regression		
		Positive (%)	Negative	OR	95% CI	*p*‐value
Age (Y)	1–4	11 (20.37)	43	1		
	4.1–6	8 (17.02)	39	6.3	0.79–50	.082
	6.1–7	6 (30)	14	4	0.41–39.35	.235
	7.1–14	9 (24.3)	28	3.7	0.41–33.52	.241
BCS	2	2 (33.3)	4	1		
	3	27 (23.08)	90	0.51	0.1–3.33	.531
	4	5 (14.71)	29	0.27	0.4–2	1.94
Lactating	Yes	19 (27.5)	50	1		.05
	No	15 (17.05)	73	2.19	0.99–4.8	
Heifer	Yes	2 (10)	18	1		.23
	No (Cows)	32 (23.36)	105	0.4	0.08–1.8	
Pregnancy	Yes	15 (19.489)	62	1		.30
	No	19 (23.5)	62	0.66	0.30–1.40	
Milk yield (l)	0–2	12 (30)	28	1		
	2.1–12	12 (24.5)	37	0.97	0.37–2.6	.96
	12.1–15	2 (6.3)	30	0.2	0.04–1	.04
	15.1–28	8 (21.6)	29	0.83	0.29–2.4	.73
Parity	No	4 (21.05)	15	1		
	1	5 (17.86)	23	0.625	0.14–2.9	.54
	2	25 (22.52)	86	1.03	0.31–34	.95
Lactation no.	0–3	22 (19.1)	93	1		.14
	4–12	12 (27.9)	31	1.84	0.81–4.86	
Trimester	1st	26 (23.4)	85	1		.13
	2nd−3rd	8 (17.02)	39	0.48	0.18–1.2	
Duration of last calving (months ago)	1–2	11 (19.3)	46	1		.52
	2.1–24	23 (21.52)	78	1.3	0.57–3	
Reproductive disorders	Yes	9 (37.5)	15	1		.03
	No	25 (18.66)	109	2.9	1.13–7.42	
Anestrous	Present	2 (66.7)	1	1		.08
	Absent	32 (20.65)	123	8.33	0.73–94.9	
Abortion	Present	2 (50)	2	1		.16
	Absent	32 (20.778)	122	4.13	056–30.5	
Retained placenta	Present	3 (25)	9	1		.67
	Absent	31 (21.23)	115	1.34	0.34–5.28	
Repeat breeding	Present	2 (33.3)	4	1		.42
	Absent	32 (11.05)	120	1.81	0.36–11.62	

#### 3.2.2 Multivariable analysis

**FIGURE 3:**
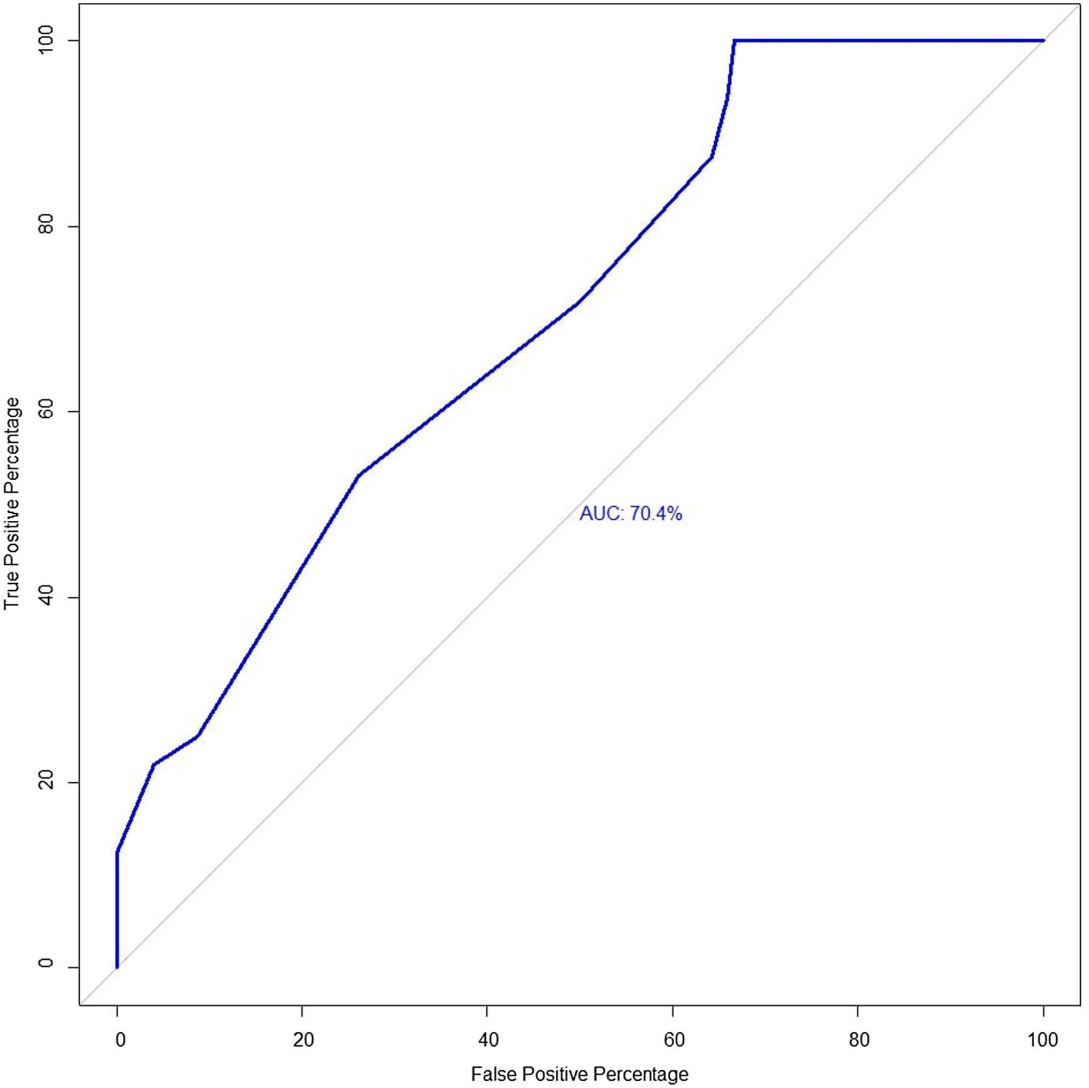
Plot of true positive percentage versus false positive percentage for a receiver operating characteristic curve (ROC) of the final multivariable logistic regression model of brucellosis in dairy cattle of CMA, Bangladesh

No two‐way interaction between the variables in the final model was significant. In the final model, cows that were lactating (OR: 2.59; 95% CI: 1.02–6.55), produced less than 2 litres of milk (OR: 29.6; 95 CI: 4.3–353.8), produced 2–12 litres of milk (OR: 4.8; 95% CI: 1.1–33.4) and cows with a history of reproductive disorders (OR: 3.2; 95% CI: 1.2–10.1) had higher odds of being seropositive for brucellosis (on parallel testing) (Table 5). The Likelihood Ratio test (LRT) of goodness of fit was not significant (*p* = .14) and the area under the receiver operating curve (ROC) was 0.70, indicating that the model fitted the data well and had a high predictive ability to discriminate seropositive and seronegative animals (Figure 3).

**TABLE 5:** Risk factors retained in the final multivariable logistic regression model for brucellosis in dairy cattle of CMA, Bangladesh

Variables	Categories	Odds ratio	95% CI	p
Reproductive disorder	No	1		
	Yes	3.2	1.2–10.1	0.034
Milk yield (l)	0–2	29.6	4.3–353.8	0.002
	2.1–12	4.8	1.11–33.4	0.058
	12.1–15	1		
	15.1–28	3.6	0.78–26.4	0.130
Lactation	No	1		
	Yes	8.1	1.1–9.5	0.034

### 3.3 Farm management practice

All six farms followed regular deworming schedules, utilized artificial insemination (AI) for breeding and did not allow their cattle to graze. Five farms had cement floors with no maternity pens and did not test the animals for brucellosis prior to introduction into the herd. Cattle on three of the farms consisted of animals that were both raised on the farm as well as animals from other sources (market, neighbour etc.). Cattle from two farms and those from the last farm were born on the same farm and obtained from another source respectively (Table 6).

**TABLE 6:** Existing farm management practices in dairy cattle in Chittagong Metropolitan City, Bangladesh (*N* = 6, *n* = 158)

Factors	Categories	Frequency	%
Types of farm	Cattle	5	83.3
	Mixed (cattle with goat/sheep)	1	16.7
Farm size	25–41	3	50.0
	52–56	3	50.0
Floor	Brick	1	16.7
	Cement	5	83.3
Maternity pen	No	5	83.3
	Yes	1	16.7
Replacement of animal	Own	2	33.3
	Others (market, neighbour etc.)	1	16.7
	Both	3	50.0
Replacement with prior testing	No	5	83.3
	Yes	1	16.7
Breeding system	AI	6	100
Bio‐security condition of farms	Good	2	33.33
	Moderate	4	66.67
Deworming	Yes	6	100
	No	0	0
Vaccination against brucellosis	Yes	1	16.67
	No	3	50
	Don't know	2	33.33
Grazing	No	6	100
	Yes	0	0
Consultancy by	Veterinarian	4	66.67
	Others (Local veterinary practitioner)	2	34.33
Presence of pet animal	No	6	100
	Yes	0	0
Fate of aborted calf	Throwing in open place or offer dog	5	83.33
	Buried	1	16.67
Knowledge about brucellosis	No	4	66.67
	Yes	2	33.33

### 3.4 Comparison of the serological test results

About 7.6% (12/158) and 1.3% (2/158) of cattle were found to be infected with acute and chronic brucellosis respectively. The relative sensitivity and specificity of the RBPT was found 85.7 and 60% respectively. The Kappa statistics value was 86% suggesting a very good agreement between the tests (*p* < .001) (Table 7).

**TABLE 7:** Outputs of Kappa statistics to assess the agreement between RBPT and cELISA and the test characteristics of RBT

RBPT	cELISA		Total	Kappa statistic
	Positive	Negative		
Positive	12 (Acute infection)	20 (Probable false positive)	32	Agreement = 86% *p* < .000
Negative	02 (Chronic infection)	124	126	
Total	14	144	158	
Relative Sensitivity	85.7%			
Relative Specificity	60.0%			

## 4 DISCUSSION

Our estimated seroprevalence of 8.9% (cELISA) was in agreement with the 7.6% seroprevalence of brucellosis reported using the indirect ELISA (iELISA) in commercial dairy cattle of Chittagong District (Sikder et al., 2012) and the 8.5% seroprevalence reported using the rapid *Brucella* antibody test kit in the Sirajgonj District of Bangladesh (Belal & Ansari, 2013). The results were also consistent with previous country‐wide estimates of 2.4%–8.4% made using the iELISA (Rahman et al., 2006).

The seroprevalence we reported based on the RBPT assay (20.3%) was consistent with the 20.4% reported on a government dairy farm in Dhaka, Bangladesh (Rahman et al., 2019). Rahman and Mia (1970) also reported 18.4% (95% CI: 14.8%–22.5%) seroprevalence of brucellosis in cattle using a tube agglutination test (TAT) in Mymensingh, Bangladesh. Other authors reported 2.66% to 5% seroprevalence of cattle‐level brucellosis from different parts of Bangladesh (Nahar & Ahmed, 2009; Rahman et al., 2011) using conventional serological tests. Of note, Rahman et al., (2019) recorded a true seroprevalence of bovine brucellosis as 0.6% in Mymensingh region. Factors that might contribute to the variation in seroprevalence described across studies include using different study design, sampling methods and diagnostic tests, as well as the effects of variation in climate and management systems between farms. From the above discussion, it can be noted that seroprevalence of brucellosis varied from region to region within Bangladesh.

Lactating animals were significantly associated with a higher risk of being seropositive to brucellosis both in univariable logistic regression and multivariable logistic regression analyses. Separate study from Ethiopia and Uganda reported, seropositivity was found in lactating and pregnant cows only (Adugna, Agga, & Zewde, 2013; Bugeza et al., 2019). In the non‐lactating group, there were some heifers. Sexually mature and pregnant cows are thought to be more susceptible to brucellosis than sexually immature cattle of either sex (Adugna et al., 2013). This pattern might have been attributed to the affinity of this bacterial pathogen to the pregnant uterus, to erythritol in fetal tissue, and possibly to steroid hormones that are higher during pregnancy (Barbier et al., 2017).

In our study, the odds of seropositivity increased as milk production decreased. This finding was expected as one of the clinical signs of infection with brucellosis is that cows may have lower milk production. Decreased milk production is also associated with various diseases of the reproductive tract. Therefore, the seropositive cows might also have been suffering from different reproductive diseases like metritis or endometritis from the last parturition that led to physical problems, resulting in lower milk production (Patel et al., 2014). The last trimester of gestation when the cow reached the last stage of lactation (i.e. less milk production) is a likely period for *Brucella* to infect the host (Islam, Khatun, Werre, Sriranganathan, & Boyle, 2013; Xavier, Paixão, Poester, Lage, & Santos, 2009).

Our study found that cows with reproductive disorders were more likely to be brucellosis seropositive than those without reproductive disorders and this is aligned with findings from other studies. Previous studies have found that reproductive diseases in general (Ullah et al., 2019) as well as the specific disorders: repeat breeding (Jain, Kumar, Chaturvedi, Roy, & Barbuddhe, 2019; Patel et al., 2014), retained fetal membranes (Dirar, Nasinyama, & Gelalcha, 2015; Patel et al., 2014; Sikder et al., 2012) and abortion (Chand & Chhabra, 2013; Jain et al., 2019; Matope et al., 2011; Patel et al., 2014; Sikder et al., 2012) have all been significantly associated brucellosis.

In our study, only female and cross breed animals were included; so no comparisons could be made between sexes and breeds. Higher seroprevalence of brucellosis in female and cross breed animals had also been reported by various studies (Joseph, Oluwatoyin, Comfort, Judy, & Babalola, 2015; Terefe, Girma, Mekonnen, & Asrade, 2017).

As cELISA is based on the specific epitopes of the (O‐polysaccharide), it can therefore eliminate some of the cross‐reaction and false negative problems seen in other serological tests.

We found that the RBPT and cELISA results agreed for 86% of the sampled. Previous studies that also compared RBPT with cELISA found agreement in 97% of samples (Ahasan et al., 2010; Rahman, 2015). The sensitivity was within the range (70.6%–97.7%) reported by Rahman (2015); however, the specificity was lower than the range (84.3%–99.9%) reported in that study for RBPT. The low sensitivity and specificity are not surprising (Kanani, 2007) as the RBPT cannot differentiate antibodies originating from infection with other gram negative organisms (Ducrotoy, Conde‐Álvarez, Blasco, & Moriyón, 2016) and it is known to give false negative results in early stage of infection, or immediately after abortion (Mohammed, 2013).

In this study, 32 samples were positive by RBPT, 12 of them were positive by cELISA. In contrast, two samples that were negative by RBPT were positive by cELISA. RBPT is able to detect IgM and IgG, whereas cELISA can only detect IgG. Thus, the difference in the results may be either due to the test sensitivity and specificity or due to the stage of infection (Rahman et al., 2019). The simultaneous presence of IgM and IgG in a sample suggests acute brucellosis, whereas, IgG alone suggests chronic infection with brucellosis (Godfroid, Nielsen, & Saegerman, 2010). Thus our results indicate that acutely infected animals predominate in the population and they are the likely reservoirs for spreading the disease. We recommend culling the acutely infected animals to decrease the spread of the disease in populations and thereby the risk of human brucellosis. None of the serological tests available for the diagnosis of brucellosis is recommended alone due to their imperfect sensitivity and specificity. However, simultaneous use of two tests, one IgG and another IgM detecting, and their serial interpretation (one animal is considered infected if positive in both tests) increases specificity (decreases false positive results) and thereby the positive predictive value (Rahman et al., 2019). Hence, the use of RBPT and cELISA test together and their serial interpretation can be recommended for culling decisions in our scenario.

We investigated the knowledge level among farmers regarding brucellosis infection, transmission, control and prevention. Our study suggests that most of the people that responded the questionnaire were not aware of brucellosis, which was also observed by Sikder et al. (2012). Knowledge of a disease is a crucial step in the development of prevention and control measures (Gumi et al., 2011). In the present study, majority of livestock keepers (83.3%) were not aware of brucellosis and its zoonotic potential. This lack of knowledge means that it is likely that farmers do not take required precautions when handling *Brucella* infected animals, animal products and animal by‐products. Moreover, with these results, it is certainly that no precaution was taken to prevent spread of the disease to other herds within or outside the study area. The perception that brucellosis can be cured and the habit of selling diseased animals either to the market or other livestock keepers can lead to propagation of the disease to other areas or herds which are not infected (Holt et al., 2011).

The livestock keepers that participated did not separate animal(s) that abort from their other animals. They also were not aware of any potential modes of transmission of diseases from animals to people, except through direct contact with aborted calves and tissues. As a result of this lack of awareness, workers continue to participate in high‐risk behaviours, including home slaughter of cattle and subsequent meat preparation (Lindahl, Sattorov, Boqvist, & Magnusson, 2015).

The majority of participants reported that they fed aborted fetuses to stray dogs or threw aborted materials into water canals used by small ruminants and other livestock for drinking or bathing. Dogs have been suggested to act as mechanical vectors while they drag aborted materials over the ground and increase the area where the bacteria is spread (Aparicio, 2013). The relationship of dogs and outbreak of brucellosis in cattle has also been demonstrated earlier (Wareth et al., 2017). Contamination of the water may increase the risk of disease transmission to people and other animal populations in that use those water sources (Wael, Tayel, Eltholth, & Guitian, 2010).

Strict biosecurity, restriction of animal movement and vaccination are suggested as effective control of brucellosis (Rahman, 2015). Additionally, strain 19 and RB51 vaccines are commonly used to protect cattle against infection and abortion (Dorneles et al., 2015); however, in Bangladesh, vaccination is not recommended in cattle reared under small‐scale dairy and subsistence/backyard management system due to very low seroprevalence (Rahman et al., 2019). However, in high within‐herd seroprevalence scenario, mass vaccination (avoiding pregnant animals in mid‐gestation) could be the most effective and practical method for Bangladesh.

As the brucellosis is endemic in Bangladesh, we recommend that a national control strategy be developed. In preparation for this, further research should be done to assess the impact of brucellosis on the livestock economy, livestock health and human health. Additionally, the national veterinary service must be strengthened to carry out the strategy, which includes increased collaboration between public health and veterinary services. Further, simulations of the costs associated with various control or eradication strategies must be evaluated to support this strategy.

Due to the small sample of farms in the study area, inter‐farm transmission factors and farm‐level variables (usual management practices in aborted cases, rearing other animals in to the farm etc.) were not investigated by statistical models. These are known to be important for brucellosis spread and maintenance in a farming system (Addis & Desalegn, 2018) and further research to characterize these risk factors is recommended. Other limitations of our study were that we only included female animals although male cattle can be infected with *Brucella* and play an active role in its transmission and not all cattle on a farm could be sampled due to difficulties in restraining and handling (lack of facilities at farms).

## 5 CONCLUSION

This study suggests that acute brucellosis is more frequent in the dairy cows of the study area. Cows that have reproductive disorders and are producing little milk should be prioritized for brucellosis screening in CMA. The screening tests should be used to control brucellosis in cattle in order to protect animal welfare, human health, and to minimize the economic losses. Moreover, culling of the acutely infected animals will decrease the spread of the disease in populations and thereby the risk of human brucellosis.

## CONFLICT OF INTEREST

The authors declare no conflict of interest.

## AUTHOR CONTRIBUTION

**Shariful Islam:** Conceptualization; Data curation; Formal analysis; Investigation; Methodology; Writing‐original draft; Writing‐review & editing. **Shama Ranjan Barua:** Methodology; Writing‐review & editing. **Shahnaj Parvin Moni:** Data curation; Methodology; Resources; Writing‐review & editing. **Ariful Islam:** Writing‐review & editing. **A.K.M. Anisur Rahman:** Project administration; Validation; Writing‐review & editing. **Sharmin Chowdhury:** Conceptualization; Funding acquisition; Project administration; Supervision; Writing‐review & editing.

### PEER REVIEW

The peer review history for this article is available at https://publons.com/publon/10.1002/vms3.348.

## Supporting information

AppendixS1
