# Supplementary material for: Seroprevalence and risk factors for bovine brucellosis in the Chittagong Metropolitan Area of Bangladesh
Source: Vet Med Sci. 2020 Sep 19;7(1):86–98. doi: 10.1002/vms3.348 (PMC7840193; doi:10.1002/vms3.348)
Supplement: Supplementary file 1 — AppendixS1 [file VMS3-7-86-s001.docx]

**Appendix-1**

**Chittagong Veterinary and Animal Sciences University**

Faculty of Veterinary Medicine

Khulshi, Chittagong-4225, Bangladesh

**Verbal consent of the participant**

**Project Title:** Seroprevalence and spatial pattern of Bovine brucellosis of Chittagong Metropolitan Area

I am Dr. Shariful Islam, from Chittagong Veterinary and Animal Sciences University

Khulshi, Chittagong-4225, Bangladesh. I am a master’s student under the department of Medicine and Surgery. I am conducting a research study on “Seroprevalence and spatial pattern of Bovine brucellosis of Chittagong Metropolitan Area”. The research will help us to understand the seroprevalence and risk factors for brucellosis in dairy cattle. I request you to participate in an interview with a pretested questionnaire and allow us to collect biological samples from your dairy cattle. Your participation and allowing to collect samples is voluntary. If you do not wish to participate, you may stop at any time. Responses will be completely anonymous; your name will not appear anywhere in the final write up. There are minimal risks for the animals while sampling. If at any time during our questionnaire completion and sampling you find uncomfortable and harmful for your animals let me know, and we will not continue our activities. I will do everything I can to protect you and your animal’s health security and privacy.

We will provide a copy of this letter for your records. If you have any further inquiries and information you need to know about the research, please contact Dr. Sharmin Chowdhury, faculty of veterinary medicine, Chittagong Veterinary and Animal Sciences University

Khulshi, Chittagong-4225, mobile number: +8801554331355, email id: [sharminchowdhury77@gmail.com](mailto:sharminchowdhury77@gmail.com).

Now, I would like to start our sampling activities if you agree to participate in this study.
